# Trends in Antimicrobial Resistance of Staphylococcus aureus Uropathogens: A Retrospective Study From Duhok Province, Kurdistan Region, Iraq

**DOI:** 10.7759/cureus.77553

**Published:** 2025-01-16

**Authors:** Nawfal R Hussein, Ibrahim A Naqid, Nashwan Ibrahim, Seepal I Ahmad, Shameran S Daniel, Ali H Yahya, Abdulwahab S Muhammed, Gaylan J Muhammed, Noor T Ramadan, Sndi I Mihe, Yousif S Yousif, Delovan S Mahfodh

**Affiliations:** 1 Department of Biomedical Sciences, College of Medicine, University of Zakho, Duhok, IRQ; 2 Department of Surgery, College of Medicine, University of Duhok, Duhok, IRQ; 3 Emergency Department, Azadi Teaching Hospital, Directorate of Health, Duhok, IRQ; 4 Department of Basic Sciences, College of Dentistry, University of Duhok, Duhok, IRQ; 5 Department of Medical Education Development, College of Medicine, University of Zakho, Duhok, IRQ; 6 Department of Medicine, College of Medicine, University of Duhok, Duhok, IRQ

**Keywords:** antimicrobial sensitivity, azadi teaching hospital-duhok, iraq, staphylococcus aureus, urine samples

## Abstract

Background

*Staphylococcus aureus* is a gram-positive bacterium causing various infections, colonizing various human body sites, and leading to a wide spectrum of diseases. Antimicrobial resistance presents a growing and significant danger to worldwide public health, demanding urgent action from both governmental bodies and communities.

Objectives

This study aimed to analyze the antibiotic sensitivity of *S. aureus *for common antibiotics isolated from urine samples collected from participants at Azadi Teaching Hospital in Duhok, Iraq.

Methods

This study was conducted between January 2018 and February 2022. During this five-year period, a total of 396 samples were gathered from patients referring to Azadi Teaching Hospital, Duhok, Iraq. *S. aureus* strains were confirmed and tested in terms of susceptibility to various antibiotics using the VITEK 2 system (bioMérieux, Inc., Durham, NC).

Results

*S. aureus* isolates from urine samples from 2018 to 2022 showed the highest resistance rate in 2022. The highest resistance rate was reported for erythromycin (324, 81.82%) while the highest sensitivity rate was found for levofloxacin (275, 69.4%). Additionally, gender-based differences in antimicrobial susceptibility were negligible (p > 0.05), but generally, males had a slightly higher resistance rate compared to females.

Conclusion

According to the results, *S. aureus* isolated from urine samples varied in terms of the antibiotic susceptibility pattern with high resistance rates to erythromycin, penicillin, and oxacillin, while showing high sensitivity rates to levofloxacin, nitrofurantoin, and vancomycin. Further studies are needed with a broader geographical involvement to investigate the antibiotic sensitivity pattern in Iraq.

## Introduction

*Staphylococcus aureus *is a commensal gram-positive bacterium that was first discovered in the 1880s [1,2]. *S. aureus *causes a variety of community and hospital-acquired infections and colonizes various areas of the body [3,4]. Asymptomatic human carriers serve as the primary natural reservoirs, with the primary site of colonization being the frontal section of the nasal mucosa. Persistent nasal colonization is observed in approximately 30% of the human population and is regarded as a key predisposing factor in disease pathogenesis [5].

Urinary tract infection (UTI) denotes the infiltration of the urinary tract by one or multiple uropathogenic bacterial species and it is one of the most prevalent bacterial illnesses resulting in notable bacteriuria and the manifestation of symptoms such as dysuria [6,7]. Recent research in Iraq revealed that among gram-negative isolates, *Escherichia coli* was the predominant uropathogen, while *Staphylococcus* species were the primary gram-positive bacteria responsible for causing UTIs [8]. *S. aureus *was responsible for about 13.8% of Duhok province cases of UTIs [9] and it significantly contributes to rising healthcare costs and affects around 150 million people annually worldwide [10,11].

Antibiotic resistance poses a significant public health issue. The onset of antibiotic resistance in *S. aureus *was initially documented in the mid-1940s. The increased use of antimicrobial agents in the community without medical prescription, coupled with their affordability, is believed to be a contributing factor to the development and the spread of antimicrobial resistance [12,13]. In the Kurdistan region of Iraq, there is a notable absence of surveillance data in general medical practice, resulting in an insufficient understanding of resistance rates in *S. aureus* uropathogens. This project aimed to study the antibiotic sensitivity of *S. aureus *for common antibiotics isolated from urine samples over the period of five years between 2018 and 2022.

## Materials and methods

Study design

This study was performed between January 2018 and February 2022. During this five-year period, a total of 396 samples were gathered from patients referring to Azadi Teaching Hospital, Duhok. The patients were within the age range of eight to 71 years from both genders (315 females and 81 males).

Sample collection and processing

Clean catch midstream urine samples were collected from participants attending outpatient departments at Azadi Teaching Hospital in Duhok, Iraq. Urine samples (4-5 ml) were collected in sterile disposable containers and promptly transferred to the laboratory of the same hospital.

Urine samples were collected and processed following aseptic techniques. We first performed a macroscopic examination for color, turbidity, and pH. Then, microscopic wet-mount analysis was performed to detect significant bacteriuria (≥20 bacteria/high-power field (HPF)) and pyuria (≥10 pus cells/HPF). Initial cultures were done on nutrient agar, MacConkey agar, and blood agar and incubated at 37°C overnight to ensure reliable growth and then sub-cultured on mannitol salt agar. The cultures were then incubated overnight at 37°C (Laboratory Incubator, Esco Micro Pte. Ltd., Singapore) and examined for the presence of bacterial growth.

Results were interpreted based on colony counts, with significant growth defined as ≥10⁵ CFU/mL to differentiate infection from contamination or colonization. Samples with insignificant bacterial growth (<10⁵ CFU/mL) were excluded from the study.

The inclusion criteria included male and female patients with positive microbiological evidence of UTIs, and agreement to be recruited in the present study. The exclusion criteria included patients outside the age range of eight to 71 years, patients who did not have positive microbiological evidence of UTIs, and individuals who declined to participate or did not provide consent for inclusion in the study.

*S. aureus* identification and antibiotic sensitivity test

Bacteriological techniques were used to isolate *S. aureus. *The plates were inspected for distinctive phenotypic features, including the presence of golden colonies, and subjected to gram staining. Colonies exhibiting mannitol fermentation were selected and subjected to additional sub-culturing on mannitol salt agar using the streak plate method to ensure the acquisition of a pure culture. Further identification of presumptive *S. aureus* colonies was carried out through biochemical assays, including catalase and agglutination tests [14].

Antimicrobial susceptibility testing (AST) was performed utilizing the VITEK 2 system (bioMérieux, Inc., Durham, NC) for the determination of antibiotic susceptibility test by the way of transformation of a limited number of colonies from the culture of *S. aureus *that was pure and fresh into a transparent plastic test tube comprising 3.0 mL of sterile saline (aqueous 0.45% to 0.50% sodium chloride (NaCl), pH 4.5 to 7.0) to prepare a suspension (0.5 McFarland standard). The integrated vacuum apparatus was used for the vaccination of the ID-GN card (bioMérieux) by a suspension of microorganisms. Test tubes (comprising of microorganism suspension) were set into a cassette (special rack) and the identity (ID) cards were set in the adjacent space. The vacuum chamber station received the cassette manually. After the vacuum had been applied and air had once again been introduced into the station, the test wells were filled by the suspension of microorganisms that were forced throughout the conveyed tube into microchannels. During the run, *S. aureus* American Type Culture Collection (ATCC) 29213 was used as a control strain to ensure quality assurance.

Ethical approval

The study procedure and informed consent forms were approved by the Ethical Committee, College of Medicine, University of Zakho, Kurdistan Region, Iraq. Written informed consent was acquired from each subject before sampling.

Statistical analysis

GraphPad Prism version 8 (GraphPad Software, LLC, San Diego, CA) was used for statistical analysis. The results were calculated for categorical data as numbers and percentages. The significant differences between sensitivity and resistance were calculated using the chi-square test. P-value <0.05 was considered significant.

## Results

Trend of antimicrobial resistance among participants

**Figure 1:**
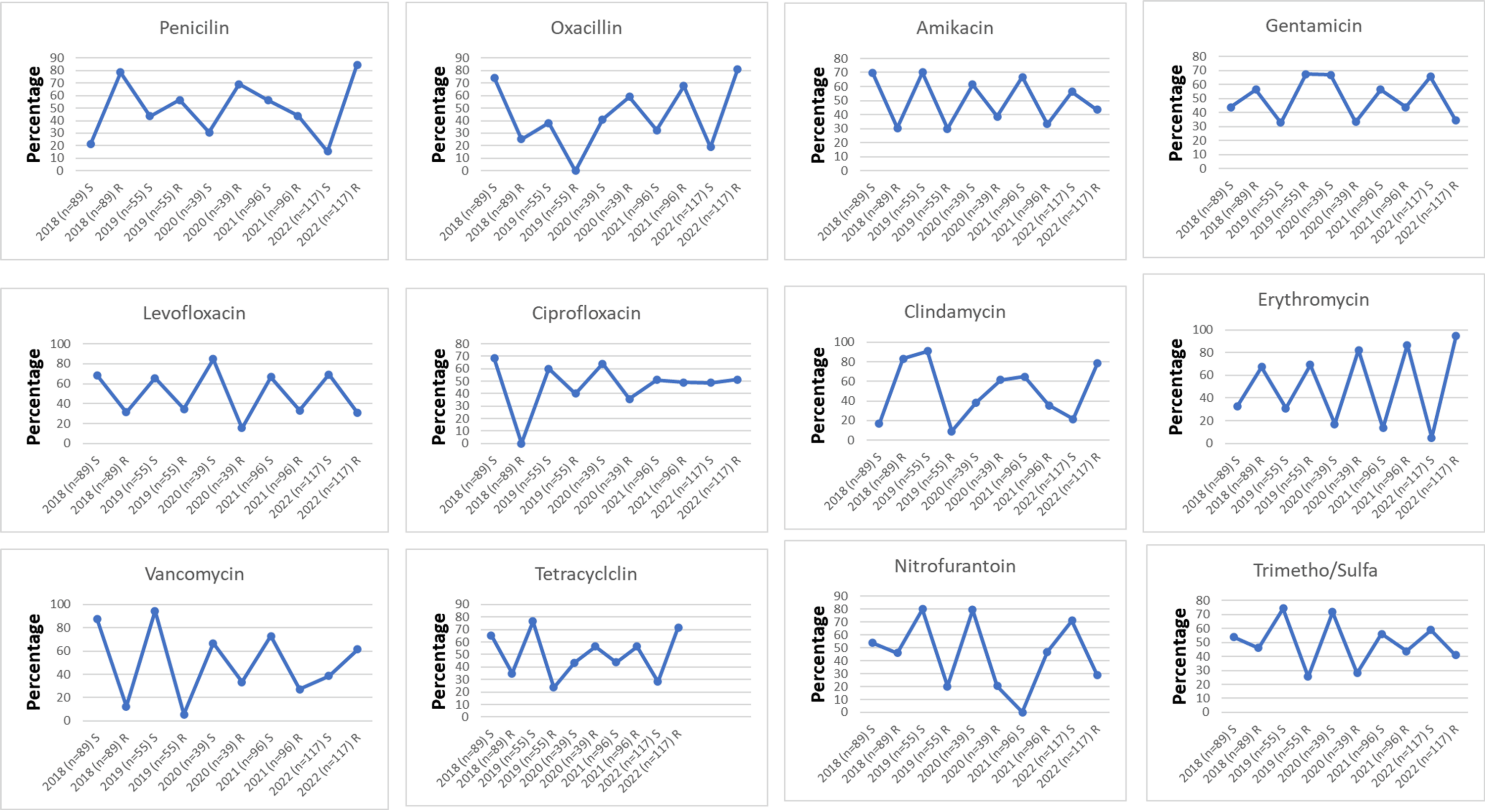
Trend of antimicrobial susceptibility profile of S. aureus isolates from urine samples over a five-year period.

Regarding the antimicrobial susceptibility pattern of *S. aureus *isolates from urine samples, our results showed that penicillin’s highest resistance percentage was in 2022, which was 99 (84.6%). For oxacillin, the resistance peaked at 95 (81.2%) in 2022, with the lowest rate recorded in 2018 at 23 (25.2%). Gentamicin and levofloxacin reached their highest resistance rates in 2019 at 37 (67.3%) and 19 (34.5%), respectively. Amikacin’s highest resistance rate was 51 (43.6%) in 2022 and the lowest was 16 (29.9%) in 2019. Ciprofloxacin had its highest resistance at 60 (51.3%) in 2022 and the lowest at 28 (31.5%) in 2018. Clindamycin’s resistance peaked at 74 (83.1%) in 2018, dropping to its lowest at five (9.1%) in 2019. Erythromycin, over the five years, hit its highest resistance at 111 (94.9%) in 2022 and its lowest at 60 (67.4%) in 2018. Tetracycline’s highest resistance was in 2022 at 84 (71.8%), with the lowest recorded in 2019 at 13 (23.6%). Vancomycin’s resistance peaked at 72 (61.5%) in 2022, while the lowest was three (5.5%) in 2019. Nitrofurantoin had its highest resistance at 45 (46.9%) in 2021, and the lowest at 11 (20%) in 2019. Trimethoprim reached its highest resistance in 2018 at 41 (46.1%), with the lowest at 14 (25.5%) in 2019 (Figure 1).

Overall resistance rate of antibiotics

The total sensitivity profile of *S. aureus *isolated from urine samples is shown in Table 1. We found that 324 (81.82%), 269 (67.9%), and 240 (60.1%) of the isolated *Staphylococcus aureus *were resistant to erythromycin, penicillin, and oxacillin, respectively. However, the isolated *S. aureus *was highly sensitive to levofloxacin (275, 69.4%), vancomycin (271, 68.43%), and nitrofurantoin (257, 64.9%) (Table 1).

**Table 1:** Overall antimicrobial susceptibility patterns of S. aureus isolated from urine samples.

Antibiotic susceptibility pattern		
Antibiotics	Resistant rate, No. (%)	Sensitivity rate, No. (%)
Penicillin	269 (67.9)	127 (32.1)
Oxacillin	240 (60.6)	156 (39.4)
Gentamicin	182 (46.1)	213 (53.9)
Amikacin	141 (35.6)	255 (64.4)
Levofloxacin	121 (30.6)	275 (69.4)
Ciprofloxacin	171 (43.2)	225 (56.8)
Clindamycin	170 (42.9)	226 (57.1)
Erythromycin	324 (81.82)	72 (18.18)
Vancomycin	125 (31.57)	271 (68.43)
Tetracycline	204 (51.52)	192 (48.48)
Nitrofurantoin	139 (35.10)	257 (64.90)
Trimethoprim	156 (39.39)	240 (60.61)

Antimicrobial sensitivity profile of *S. aureus *according to gender

In terms of antimicrobial susceptibility patterns of *S. aureus *isolated from urine samples according to gender, no significant difference was detected between males and females (p > 0.05). In males, the highest resistance percentages were observed for erythromycin (68, 84%) and penicillin (55, 67.9%), whereas in females, it was for erythromycin (256, 81.3%) and penicillin (214, 67.9%) (Table 2).

**Table 2:** Antimicrobial susceptibility patterns of S. aureus isolated from urine samples according to gender. The p-value is determined using the chi-square test.

Antibiotics	Frequency of isolates from urine, No. (%)				P-value	Chi-square value
	Male (n = 81)		Female (n = 315)			
	Sensitive	Resistant	Sensitive	Resistant		
Penicillin	26 (32.1)	55 (67.9)	101 (32.1)	214 (67.9)	0.99	1
Oxacillin	31 (38.3)	50 (61.7)	125 (39.7)	190 (60.1)	0.81	0.05
Gentamicin	38 (46.9)	43 (53.1)	175 (55.7)	139 (44.3)	0.15	2
Amikacin	54 (66.7)	27 (33.3)	201 (63.8)	114 (36.2)	0.63	0.2
Levofloxacin	52 (64.2)	29 (35.8)	223 (70.8)	92 (29.2)	0.28	1
Ciprofloxacin	42 (51.9)	39 (48.1)	183 (58.1)	132 (41.9)	0.31	1
Clindamycin	44 (54.3)	37 (45.7)	182 (57.8)	133 (42.2)	0.58	0.3
Erythromycin	13 (16.0)	68 (84.0)	59 (18.7)	256 (81.3)	0.57	0.3
Vancomycin	55 (67.9)	26 (32.1)	216 (68.6)	99 (31.4)	0.89	0.1
Tetracycline	38 (46.9)	43 (53.1)	154 (48.9)	161 (51.1)	0.75	0.1
Nitrofurantoin	50 (61.7)	31 (38.3)	207 (65.7)	108 (34.3)	0.51	0.4
Trimethoprim	50 (61.7)	31 (38.3)	190 (60.3)	125 (39.7)	0.72	0.1

## Discussion

It was previously found that Staphylococcus species were the main gram-positive bacteria accountable for UTIs, responsible for about 13.8% of Duhok province cases of UTIs, with antimicrobial resistance among urinary pathogens being a global concern [8,9]. Therefore, this study aimed to investigate the frequency of S. aureus microorganisms responsible for UTIs and their antimicrobial susceptibility patterns in the general population in Duhok province, Iraq.

In the present study, a total of 396 urine samples were collected from patients seeking medical care at Azadi Teaching Hospital in Duhok province. Our results showed that in 2018, the highest percentage of resistance was recorded by clindamycin (74, 83.1%), while vancomycin (78, 87.6%) was the most sensitive. In 2019, the most resistant ones were erythromycin (38, 69.1%) and gentamicin (37, 67.3%), while vancomycin (3, 5.5%) was the least resistant one. In 2020, again, erythromycin (32, 82.1%) was the most resistant, and levofloxacin (33, 84.6%) was the most sensitive. In 2021, the highest resistance percentage was for erythromycin (83, 86.5%), and the lowest resistance percentage was for vancomycin (26, 27.1%). In 2022, the highest resistance percentage was for erythromycin (111, 94.9%), and the highest sensitivity percentage was for nitrofurantoin (83, 70.9%). Overall, the year 2022 had the highest percentage of antibiotic resistance (822, 58.51%). This increase in antibiotic resistance can be a part of the global increase in antibiotic resistance rate. Such a global increase in antibiotic resistance may be attributed to widespread misuse of antibiotics due to over-the-counter availability of such drugs, lack of awareness, or regulatory enforcement [15,16]. Other factors include excessive use of antibiotics in veterinary and agriculture, contamination of the environment with antibiotics from medical waste, and natural selection and genetic mutations [16]. Besides, the COVID-19 pandemic had a deleterious impact on the antibiotic resistance rate due to several factors. These factors include increased antibiotic use for COVID-19 patients, disruption of stewardship programs, and hospital overcrowding.

In the present study, it was observed that *S. aureus *isolates were highly resistant to erythromycin, penicillin, and oxacillin. The highest sensitivity rates were observed against levofloxacin, vancomycin, and nitrofurantoin. Specifically, erythromycin (324, 81.82%), penicillin (269, 67.9%), and oxacillin (240, 60.6%) recorded the highest resistance rate in the present study, which was higher than that in other studies conducted in Iraq [15]. It was previously shown that the resistance rates were 40% for erythromycin and 33.3% for oxacillin. In a study conducted in Turkey, the resistance rate was 17.7% for erythromycin and 100% for penicillin [16]. The differences in results between the current study and other studies could be due to significant differences in sample size, sampling methods, and geographical/environmental variations. The elevated levels of resistance observed to erythromycin, oxacillin, and penicillin in our study are concerning, particularly when compared to rates reported in other countries. This highlights the danger of rapid dissemination of antibiotic resistance within clinical isolates in our region and a rapid plan is needed to tackle such an issue. On the other hand, the high sensitivity rates in the present study were for levofloxacin, nitrofurantoin, and vancomycin, which were nearly similar to another study conducted in Iraq where high sensitivity was observed for levofloxacin, nitrofurantoin, and trimethoprim [15]. This high sensitivity may be due to the low availability of these antibiotics, or it may be due to their high price.

The antimicrobial susceptibility patterns of *S. aureus *isolated from urine samples were analyzed based on gender, revealing no significant differences between males and females for all antibiotics examined. Among males, the highest resistance rates were observed in erythromycin (68, 84.0%), penicillin (55, 67.9%), and oxacillin (50, 61.7%). Conversely, the highest rate of sensitivity was observed in vancomycin with more than two-thirds of the males (55, 67.9%). Similarly, among females, the highest resistance percentages were observed in erythromycin (256, 81.3%), followed by penicillin (214, 67.9%) and oxacillin (190, 60.1%), and the lowest resistance was observed in levofloxacin (92, 29.2%).

The study has several strengths and limitations. Spanning five years (2018-2022), it provides valuable insights into antimicrobial resistance trends in the country, a region with limited surveillance data. It includes 396 *S. aureus* uropathogens from both genders across a wide age range, offering a reasonable sample size for analysis. However, its focus on a single hospital limits the generalizability of findings to other regions. The retrospective design relies on existing records, which may introduce biases such as incomplete data. Additionally, the lack of molecular characterization of resistance mechanisms (e.g., resistance genes) reduces the depth of analysis. Despite these limitations, the study provides important data for addressing the antimicrobial resistance trends in the region.

## Conclusions

Our study showed that the resistance rate of most antibiotics is increasing. Regarding trends in antibiotic resistance, erythromycin, penicillin, and oxacillin exhibited notably high resistance rates; conversely, levofloxacin, nitrofurantoin, and vancomycin showed good sensitivity rates. A rapid robust plan is needed to tackle the issue of antibiotic resistance, promote the rational use of antibiotics, develop and enforce stewardship programs, enhance infection prevention and control programs, and raise awareness.
